# Associations Among Web-Based Civic Engagement and Discrimination, Web-Based Social Support, and Mental Health and Substance Use Risk Among LGBT Youth: Cross-Sectional Survey Study

**DOI:** 10.2196/46604

**Published:** 2023-06-26

**Authors:** Xiangyu Tao, Celia Fisher

**Affiliations:** 1 Department of Psychology Fordham University Bronx, NY United States; 2 Center for Ethics Education Fordham University Bronx, NY United States

**Keywords:** lesbian, gay, bisexual, and transgender or nonbinary, LGBT adolescents, social media, discrimination, social support, mental health, substance use

## Abstract

**Background:**

Social media use is ubiquitous among lesbian, gay, bisexual, and transgender or nonbinary (LGBT) adolescents. The time spent on LGBT sites and involvement in social justice–oriented web-based civic activities can increase exposure to heterosexist and transphobic posts, resulting in increases in depression, anxiety, and substance use. Collaborative social justice civic engagement may also increase LGBT adolescents’ social support on the web, which may buffer the mental health and substance use risks associated with web-based discrimination.

**Objective:**

Drawing on the minority stress and stress-buffering hypotheses, this study aimed to test time spent on LGBT sites, involvement in web-based social justice activities, the mediating effect of web-based discrimination, and the moderating effect of web-based social support on mental health and substance use.

**Methods:**

An anonymous web-based survey conducted from October 20 to November 18, 2022, analyzed data from 571 respondents (mean age 16.4, SD 1.1 years): 125 cisgender lesbian girls, 186 cisgender gay boys, 111 cisgender bisexual adolescents, and 149 transgender or nonbinary adolescents. Measures included demographics, web-based LGBT identity disclosure, hours per week spent on LGBT social media sites, engagement in web-based social justice activities (Online Civic Engagement Behavior Construct), exposure to web-based discrimination (Online Victimization Scale), web-based social support (adapted from scales examining web-based interactions), depressive and anxiety symptoms, and substance use (the Patient Health Questionnaire modified for Adolescents; Generalized Anxiety Disorder 7-item; and Car, Relax, Alone, Forget, Friends, Trouble Screening Test).

**Results:**

The time spent on LGBT social media sites was unrelated to web-based discrimination after civic engagement was accounted for (90% CI −0.007 to 0.004). Web-based social justice civic engagement was positively associated with social support (β=.4, 90% CI 0.2-0.4), exposure to discrimination (β=.6, 90% CI 0.5-0.7), and higher substance use risk (β=.2, 90% CI 0.2-0.6). Consistent with minority stress theory, exposure to web-based discrimination fully mediated the positive association between LGBT justice civic engagement and depressive (β=.3, 90% CI 0.2-0.4) and anxiety symptoms (β=.3, 90% CI 0.2-0.4). Web-based social support did not moderate the association between exposure to discrimination with depressive (90% CI −0.07 to 0.1) and anxiety symptoms (90% CI −0.06 to 0.1) and substance use (90% CI −0.04 to 0.01).

**Conclusions:**

This study highlights the importance of examining LGBT youth’s specific web-based activities and the need for future research to focus on the intersectional experiences of LGBT adolescents from racial and ethnic minoritized groups through culturally sensitive questions. This study also calls for social media platforms to implement policies that mitigate the effects of algorithms that expose youth to heterosexist and transphobic messaging, such as adopting machine learning algorithms that can efficiently recognize and remove harmful content.

## Introduction

### Background

Social media is a part of daily life for most US adolescents [1], and lesbian, gay, bisexual, and transgender or nonbinary (LGBT) youth spend more time on social media than their peers [2]. Social media can be a powerful tool for facilitating social support networks among LGBT youth. Social media features, such as the ability to connect people from different geographic locations and the ability to join groups or communities based on shared interests, experiences, and identities, can create opportunities for LGBT youth to interact with others who share similar experiences and identities, providing a sense of belonging and social support [3]. This is particularly important for LGBT youth, who may struggle to find supportive peers and mentors in their offline social environments because of the stigma and discrimination they may face [4]. Web-based communities can offer a safe and supportive space for these youth to connect with others who understand their experiences, reducing feelings of isolation and providing a source of emotional support [5,6]. Accordingly, empirical research indicates that more time spent on social media substantially increases LGBT youth’s engagement with other queer persons on LGBT social media sites [2,3,7]. These sites can provide “safe spaces” where youth feel supported in counter heteronormative environments in ways that support mental health [2,3,7]. Social support on these sites can be bidirectional, characterized by both giving and receiving support from friends in the web-based LGBT community [8]. However, in a recent national survey, LGBT adolescents self-reported both positive (96%) and negative (88%) effects of social media on their mental health and well-being [9,10]. Longer time spent on social media has been associated with greater mental health risk among general adolescent populations [11], specifically adolescents from racial and ethnic minoritized groups [12]. Although LGBT populations face unique and disproportionate physical and mental health disparities compared with their heterosexual and cisgender peers [13-15], social media has the potential to exacerbate these disparities by exposing LGBT youth to heterosexist and transphobic messaging, leading to increased depression, anxiety, and substance use [9,10]. For youth visiting LGBT sites, exposure to heterosexist and transphobic posts may be increased by individuals specifically looking to target these youth. Exposure to discrimination can also be a consequence of social media algorithms that use profile information and interests to sort content that the algorithm is programmed to assume users are likely to want to see, not necessarily distinguishing between positive and negative posts about susceptible populations [16].

To date, most research has focused on the time spent on LGBT sites involving peer communities or following LGBT celebrities [2,4]. Little is known about other types of activities on social media. Promoting social justice and civic action for the LGBT community is one such activity that has not been examined. Social media LGBT civic engagement may have potential benefits and drawbacks for LGBT youth. On the one hand, such engagement may provide an avenue for individuals to connect with others who share similar experiences [6]. In addition, web-based civic engagement may help create a sense of belonging and validation for LGBT youth, who may feel marginalized or isolated in offline settings [5]. At the same time, civic engagement may have a paradoxical effect. In its mission to promote social justice causes and combat anti-LGBT incidents or policies [17,18], such engagement increases exposure to heterosexist and transphobic web-based messages, thereby potentially increasing a sense of isolation and minority stress. Exposure to such discriminatory messaging on these sites may be especially salient during the current wake of anti-LGBT state laws [8]. Although not yet studied among LGBT youth, a study conducted during the height of the COVID-19 pandemic and the Black Lives Matter movement demonstrated that racial and ethnic web-based social justice–oriented civic engagement was associated with increased exposure to social media racial discrimination, which in turn was associated with higher mental health and alcohol use risks [12]. In this study, more than half of adolescents from racial and ethnic minoritized groups identified as LGBT reported significantly higher rates of social media use, web-based racial justice civic engagement, and depressive symptoms, underscoring the need for research on LGBT social justice civic engagement.

According to the minority stress theory, individuals who experience discrimination because of their sexual and gender minority status in society are at risk of heightened stress, potentially leading to depression, anxiety, and general psychological distress [19,20]. In recent years, these disparities among LGBT youth have become even more pronounced. In a 2021 national survey of LGBT youth [10], 75% reported experiencing discrimination based on their sexual and gender identity, with high rates of youth reporting symptoms of depression (62%), anxiety (72%), and substance use. The stress-buffering model [21] suggests that social support can help mitigate mental health risks for those experiencing minority stress. However, such relationships have largely been examined in an offline context [22]. A recent meta-analysis of youth in general showed only a small and unimportant relationship between web-based social support and depression [23]. Studies involving ethnically diverse young adults found conflicting results regarding the moderating effect of perceived web-based social support on the relationship between exposure to discrimination and mental health [24,25].

### This Study

LGBT youth face unique mental health challenges in response to their minoritized and stigmatized status in many social contexts. High social media use rates among LGBT youth expose them to web-based discrimination, which in turn has been associated with mental health concerns. This may be further exacerbated when youth engage in LGBT social justice web-based communities. At the same time, these web-based LGBT communities may provide social support that moderates the effect of anti-LGBT posts on mental health. Drawing on the minority stress and stress-buffering hypotheses, this study examined associations among LGBT social media general and social justice–oriented community engagement; web-based social support; exposure to discriminatory posts; and depression, anxiety, and substance use. We also assessed whether web-based support mitigated the relationship between exposure to discrimination and mental health.

**Figure 1:**
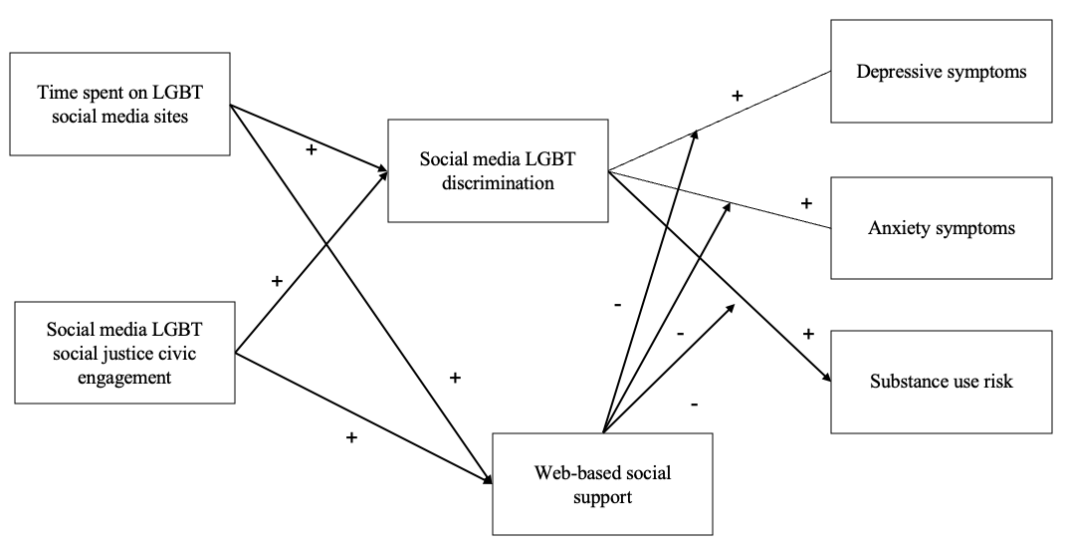
The conceptual mediated moderation model. +: positive association and −: negative association; covariates and direct associations between time spent on lesbian, gay, bisexual, and transgender or nonbinary (LGBT) social media sites and social media LGBT social justice civic engagement with mental health and substance use risk were omitted.

We hypothesized that (1) increased time spent on LGBT social media sites and higher levels of social justice civic engagement would be associated with higher levels of both social support and exposure to web-based discrimination; (2) higher levels of web-based social support for LGBT youth would be associated with lower mental health and substance use risks, whereas exposure to web-based LGBT discrimination would be associated with higher levels of mental health and substance use risks; (3) exposure to web-based discrimination would mediate the positive association between time spent on LGBT social media sites and social justice civic engagement with mental health and substance use risks; and (4) web-based social support would moderate the positive association between exposure to web-based discrimination and mental health and substance use risks (Figure 1).

## Methods

### Participants and Recruitment

Data were collected in October and November 2022. Eligible participants were middle school or high school adolescents aged 14 to 18 years who self-identified as LGBT, resided in the United States, and used social media for at least 5 days per week. Recruitment was conducted through Instagram (Meta Platforms, Inc) paid advertisements and Qualtrics Experience Management (Qualtrics International, Inc), a platform aggregating different panels that include people who registered to participate in web-based surveys. Advertisements through Instagram and Qualtrics Experience Management included a link to the screener. A total of 2203 people clicked on the link, and 879 people were eligible for the study. All the survey scale items required a forced response to prevent missing data. Participants were informed that they could exit the survey at any time, and their data would not be analyzed: 7.7% (68/879) dropped out. To prevent fraudulent and low-quality responses, those who failed the attention check questions in the main survey were excluded from the data analysis, resulting in a total of 778 adolescents completing the main survey in an average of 20.31 minutes. A manual data validity check was then conducted by excluding those with a mismatch between their self-reported date of birth and age in years, international or duplicate IP addresses, and time spent responding to <2 SDs of the average survey time, resulting in a sample of 581 participants. LGBT adolescents who were recruited through Instagram and provided valid responses were given a US $10 Amazon gift card within 3 days of completing the survey. Those who were recruited through Qualtrics Experience Management and provided valid responses were awarded equivalent points in their panel systems for gift card exchange within 7 days after completing the survey.

For this study, because only 9 respondents self-identified as questioning or unsure and 1 identified as a cisgender gay girl, they were not included in the final analysis, which resulted in a final sample of 571 participants. The participants were from 48 states, with the largest representation from the South (181/571, 31.7%) and the West (181/571, 31.7%) regions, followed by the Northeast (117/571, 20.5%) and the Midwest (91/571, 15.9%) regions. After conducting data analysis, the authors presented the results to an LGBT college student advisory board (n=6) that advised on interpreting the results.

### Ethics Approval

All procedures were approved by the Fordham University Institutional Review Board (Protocol #2239).

### Measures

#### Demographic Information

Demographic variables included age; self-reported race or ethnicity using 2 items, that is, “How do you describe your primary (secondary) race/ethnicity?” with response options “American Indian/Alaska Native,” “Arabic, Middle Eastern or North African,” “Asian (e.g. East, South, Southeast),” “Black (e.g. African American, African, Caribbean),” “Hispanic/Latinx,” “Native Hawaiian or Other Pacific Islander,” “White,” and “Other”; gender and sexual identity; grade; parent education level; financial insecurity; and household income. We asked whether adolescents had disclosed their LGBT identities on LGBT social media sites.

#### Time Spent on LGBT Social Media Sites

In addition to reporting the use of popular social media platforms during the past month (eg, Twitter [Twitter, Inc], Instagram, and TikTok [ByteDance]), respondents were also asked 2 questions regarding their use of LGBT social media community sites (although we did not ask them to name the sites). The questions asked them to indicate approximately how many days in each week and how many hours per day they spent on these sites. The average weekly use was calculated by multiplying hours by days.

#### LGBT Social Justice Civic Engagement

Web-based LGBT social justice civic engagement in the past month was assessed by adapting the language of the Online Civic Engagement Behavior Construct [26] for the general population and adolescents from racial and ethnic minoritized groups [12], specifically for LGBT youth. The scale included 3 items describing participation in civic publication (“post or share links in support of LGBT rights on social media”) and 4 items on activity coordination (“plan activities that support LGBT rights on social media”). Response options ranged from 0=“Never” to 4=“Almost daily” based on a 5-point Likert-type scale. Confirmatory factor analyses of the current data yielded a final 1-factor model with good internal consistency of a Cronbach α of .92 (for details, refer to Multimedia Appendix 1). The composite score for bivariate analyses was calculated using the average of the scores of all items.

#### Social Media LGBT Discrimination

The Online Victimization Scale [27] was adapted for LGBT youth to assess the past month’s exposure to discrimination. The original scale included 2 subscales assessing exposure to personally targeted discrimination (4 items, eg, “People have said mean or rude things about me on social media because of my LGBT identity”) and to vicarious discrimination (3 items, eg, “I have witnessed people saying mean or rude things on social media about another person’s LGBT identity”). In this study, “race and ethnicity” were replaced by “LGBT identity,” and “online” was substituted with “on social media” for all items. Responses ranged from 0=“Never” to 4=“Almost daily” based on a 5-point Likert-type scale. The scale has been used to assess adolescents’ exposure to individual and vicarious racial discrimination on social media with adequate internal consistency (Cronbach α=.77-.86) [12,28]. Confirmatory factor analyses of the current data yielded a final 1-factor model with good internal consistency of a Cronbach α of .90 (for details, refer to Multimedia Appendix 1). The composite score for bivariate analyses was calculated using the average of the scores of all items.

#### Social Support on LGBT Social Media

A 7-item scale was constructed to assess giving (3 items, eg, “If I see some LGBT friends post a social media status update that indicates they are upset, I try to post a comforting comment on their status”) and receipt of support (4 items, eg, “There are LGBT friends on social media who care about my feelings”) based on items drawn from 2 studies [29,30]. The term “Facebook” in the original items was replaced by “social media.” The response options ranged from 0=“Strongly disagree” to 5=“Strongly agree” on a 5-point Likert-type scale. Confirmatory factor analyses of the current data yielded a final 1-factor model with good internal consistency of a Cronbach α of .84 (for details, refer to Multimedia Appendix 1). The composite score for bivariate analyses was calculated using the average of the scores of all items.

#### Mental Health and Substance Use Risk

Three psychometrically validated scales were administered to adolescents. The 9-item Patient Health Questionnaire modified for Adolescents (PHQ-A) [31,32] assesses the frequency of past-month experiences with depressive symptoms (eg, “Trouble falling asleep, staying asleep, or sleeping too much”). The 7-item Generalized Anxiety Disorder Screener (GAD-7) [33,34] assesses adolescents’ experience of anxiety symptoms in the past month (eg, “Being so restless that it is hard to sit still”). Responses for PHQ-A and GAD-7 are provided on a 4‐point Likert-type scale anchored by 0=“Not at all” and 3=“Nearly every day.” The 6-item Car, Relax, Alone, Forget, Friends, Trouble Screening Test [35] was adopted to identify substance use risk only among adolescents who reported substance use in the past month (eg, “Do you ever use alcohol or drugs to relax, feel better about yourself, or fit in? [Yes or No]”). For all 3 scales, the composite scale scores were computed by adding the sum of responses to the items. All scales had good internal consistency in the current sample: Cronbach α of .92 for PHQ-A; Cronbach α of .92 for GAD-7; and Cronbach α of .88 for Car, Relax, Alone, Forget, Friends, Trouble Screening Test.

### Data Analysis

To ensure the diversity of social media experiences across different sexual and gender identities and to achieve adequate power of 0.80 for the structural equation modeling (SEM) analysis (*df*=25-60 [36]), for each of the 4 sexual orientation or gender groups, we recruited a minimum of 100 self-identified LGBT youth. The final sample consisted of 571 participants (mean age 16.37, SD 1.07 years), including 125 (21.9%) cisgender lesbian girls, 186 (32.5%) cisgender gay boys, 111 (19.4%) cisgender bisexual adolescents (100 girls and 11 boys), and 149 (26%) transgender or nonbinary adolescents (42 transgender boys, 53 transgender girls, and 54 gender nonbinary adolescents). Following confirmatory factor analyses of social media LGBT social justice civic engagement, social media LGBT discrimination, and web-based social support experiences, descriptive statistics were calculated to describe the sample demographics, including age, race and ethnicity, grade, socioeconomic status measured by financial security, household income, parental education level, social media community engagement, and mental health and substance use risk, followed by chi-square and multivariate analysis of variance (MANOVA) tests with Tukey post hoc comparison to examine differences in demographics and all studied variables based on age, LGBT identity, race and ethnicity, and their disclosure status on LGBT social media sites. As a prerequisite of the SEM analysis, bivariate correlations among demographics and all studied variables were calculated, and Bonferroni correction (*P*/*n* of predictors=.05/10=.005) was applied to determine the significance level of Pearson correlation coefficient.

To examine the research hypotheses, SEM with the maximum likelihood method was adopted with R (version 4.0.1; R Foundation for Statistical Computing) and the package *lavaan* [37,38]. Three SEM models were adopted (refer to Figure 1 for our conceptual model). The first model examined indirect paths from the time spent on LGBT sites and LGBT social justice civic engagement to mental health and substance use risks through exposure to social media LGBT discrimination. An alternative SEM model was then adopted to examine whether higher levels of exposure to web-based discrimination and associated depressive symptoms were in turn associated with more LGBT social justice civic engagement and more time spent on LGBT social media sites. The final mediated moderation model examined the moderating role of web-based social support in the relationship between exposure to web-based discrimination and mental health and substance use. Covariates included in the SEM analyses were chosen based on important associations between demographic variables and mental health and substance use risks.

With 2 exceptions, all the survey items required a forced response to prevent missing data. Two socially sensitive demographic items (household income and whether participants were out to people on the web) included the response option “I do not want to answer.” There were minimal missing data for these items—13.3% (76/571) and 1.4% (8/571), respectively. For these variables, pairwise deletion was used during correlational analysis. However, because this approach can lead to biased estimates and decreased power, full information maximum likelihood estimation was used for SEM analyses to handle any missing data. The goodness of fit indices for both primary and alternative SEM analyses included the comparative fit index (CFI), Tucker-Lewis Index (TLI), and root mean square error of approximation (RMSEA). A fit of >0.90 for the CFI and TLI and <0.06 for RMSEA were considered to indicate adequate fit [39]. The bias-corrected bootstrapping approach was adopted to test the indirect effects for statistical significance, as it is robust against the violation of normal distribution assumptions for both the sampling distribution and the indirect effect [40]. A total of 1000 resamples were drawn to estimate the SEs of the indirect effects and their 90% CIs.

## Results

### Demographic Data

Frequency, percentages, and chi-square analyses for differences in demographic data among LGBT groups are provided in Table 1. The respondents were in grades 8 to 12. Most self-identified as non-Hispanic White (279/571, 48.9%) or Black (149/571, 26.1%) and were from 48 states, with the largest representation from the South (181/571, 31.8%) and the West (181/571, 31.8%) regions, followed by the Northeast (117/571, 20.5%) and the Midwest (91/571, 15.9%) regions. More than half of the adolescents had parents with either college (164/571, 28.7%) or graduate (218/571, 38.2%) degrees. Approximately 20% (117/571) reported that they were financially insecure and 23% (130/571) reported a family income of ≤US $39,000. Most adolescents (519/571, 90.9%) were out to someone on LGBT social media sites, and those who were out to most or all people on social media were combined into 1 category (330/571, 57.8%) in later analyses.

Substantial demographic differences were found based on LGBT identities. Compared with non-Hispanic White youths (99/279, 35.5%), lower percentages of Black (16/149, 10.7%) and Hispanic or Latinx (15/55, 27.3%) youths identified as transgender or gender nonbinary. Black youths (15/149, 10.1%) self-reported that they were bisexual, compared with 17.9% (50/279) of non-Hispanic White and 38.2% (21/55) of Hispanic or Latinx youths. Significant regional differences were found; for instance, lower percentages of cisgender bisexual adolescents (11/111, 9.9%) were from the Northeast region compared with 19.2% (24/125) of cisgender lesbian girls, 25.9% (48/186) of gay boys, and 22.8% (34/149) of transgender or nonbinary youth. Parents of cisgender lesbian girls (92/125, 73.6%) and gay cisgender boys (139/186, 74.7%) were more likely to have a college degree or higher than bisexual (50/111, 45%) and transgender or nonbinary (101/149, 67.8%) adolescents. A total of 23.4% (26/111) to 24.2% (36/149) of cisgender bisexual and transgender or nonbinary adolescents did not report their household income, compared with 2.2% (4/186) to 8% (10/125) of cisgender lesbian girls and gay boys. Given this missing data rate and the significant association between financial security and household income (*r*=0.30; *P*<.001), the latter was not included in the SEM analyses, with financial insecurity used as an index of socioeconomic status.

**Table 1:** Number, percentages, and chi-square results for adolescents’ demographic characteristics and social media platforms used by lesbian, gay, bisexual, and transgender or nonbinary (LGBT) identities.

		Cisgender lesbian (n=125), n (%)	Cisgender gay (n=186), n (%)	Cisgender bisexual^a^ (n=111), n (%)	Transgender or nonbinary^b^ (n=149), n (%)	Total (N=571), n (%)	*χ*^2^ (*df*)		*P* value	
**Race and ethnicity**								73.4 (9)		
	Black	54 (43.2)	64 (34.4)	15 (13.5)	16 (10.7)	149 (26.1)			<.001	
	Hispanic or Latinx	10 (8)	9 (4.8)	21 (18.9)	15 (10.1)	55 (9.6)			N/A^c^	
	Non-Hispanic White	44 (35.2)	86 (46.2)	50 (45)	99 (66.4)	279 (48.9)			N/A	
	Other	17 (13.6)	27 (14.5)	25 (22.5)	19 (12.8)	88 (15.4)			N/A	
**Grade**								22.4 (12)		
	≤8	10 (8)	31 (16.7)	8 (7.2)	13 (8.7)	62 (10.9)			.13	
	9	11 (8.8)	20 (10.8)	12 (10.8)	12 (8.1)	55 (9.6)			N/A	
	10	26 (20.8)	29 (15.6)	19 (17.1)	35 (23.5)	109 (19.1)			N/A	
	11	40 (32)	53 (28.5)	33 (29.7)	42 (28.2)	168 (29.4)			N/A	
	12	38 (30.4)	53 (28.5)	39 (35.1)	47 (31.5)	177 (31)			N/A	
**Region**								29.3 (9)		
	Northeast	24 (19.2)	48 (25.9)	11 (9.9)	34 (22.8)	117 (20.5)			<.001	
	Midwest	13 (10.4)	20 (10.8)	24 (21.6)	34 (22.8)	91 (16)			N/A	
	South	45 (36)	50 (27)	44 (39.6)	42 (28.2)	181 (31.8)			N/A	
	West	43 (34.4)	67 (36.2)	32 (28.8)	39 (26.2)	181 (31.8)			N/A	
**Parent’s education**								59.1 (12)		
	High school graduate or less	18 (14.4)	39 (21)	49 (44.1)	32 (21.5)	138 (24.2)			<.001	
	Partial college	13 (10.4)	6 (3.2)	9 (8.1)	11 (7.4)	39 (6.8)			N/A	
	College degree	50 (40)	62 (33.3)	24 (21.6)	28 (18.8)	164 (28.7)			N/A	
	Graduate degree	42 (33.6)	77 (41.4)	26 (23.4)	73 (49)	218 (38.2)			N/A	
	Missing	2 (1.6)	2 (1.1)	3 (2.7)	5 (3.4)	12 (2.1)			N/A	
**Financial insecurity**								9.8 (6)		
	We cannot make ends meet	25 (20)	45 (24.2)	12 (10.8)	35 (23.5)	117 (20.5)			.13	
	We have just enough	61 (48.8)	78 (41.9)	55 (49.5)	63 (42.3)	257 (45)			N/A	
	We are comfortable	39 (31.2)	63 (33.9)	44 (39.6)	51 (34.2)	197 (34.5)			N/A	
**Household income (US $)**								71.9 (12)		
	≤30,999	33 (26.4)	59 (31.7)	24 (21.6)	14 (9.4)	130 (22.8)			<.001	
	31,000-50,999	29 (23.2)	63 (33.9)	24 (21.6)	34 (22.8)	150 (26.3)			N/A	
	51,000-79,999	28 (22.4)	31 (16.7)	16 (14.4)	32 (21.5)	107 (18.7)			N/A	
	≥80,000	25 (20)	29 (15.6)	21 (18.9)	33 (22.1)	108 (18.9)			N/A	
	Missing	10 (8)	4 (2.2)	26 (23.4)	36 (24.2)	76 (13.3)			N/A	
**Disclosure on LGBT social media sites**								77.5 (12)		N/A
	Not out to anyone	5 (4)	11 (5.9)	17 (15.3)	11 (7.4)	44 (7.7)			<.001	
	Only out to a select few people	51 (40.8)	86 (46.2)	30 (27)	22 (14.8)	189 (33.1)			N/A	
	Out to most people	47 (37.6)	53 (28.5)	41 (36.9)	47 (31.5)	188 (32.9)			N/A	
	Out to everyone	19 (15.2)	35 (18.8)	21 (18.9)	67 (45)	142 (24.9)			N/A	
	I do not want to answer	3 (2.4)	1 (0.5)	2 (1.8)	2 (1.3)	8 (1.4)			N/A	
**Social media platform**										
	LGBT- specific platforms	42 (33.6)	52 (28)	19 (17.1)	48 (32.2)	161 (28.2)	5.8 (3)		.12	
	Instagram	111 (88.8)	159 (85.5)	90 (81.1)	123 (82.6)	483 (84.6)	2.2 (3)		.53	
	TikTok	112 (89.6)	164 (88.2)	84 (75.7)	91 (61.1)	451 (79)	49.6 (3)		<.001	
	Facebook	94 (75.2)	162 (87.1)	45 (40.5)	76 (51)	377 (66)	79.1 (3)		<.001	
	Snapchat	96 (76.8)	118 (63.4)	77 (69.4)	79 (53)	370 (64.8)	20.4 (3)		<.001	
	Twitter	81 (64.8)	115 (61.8)	48 (43.2)	82 (55)	326 (57.1)	7.3 (3)		.63	
	Reddit	53 (42.4)	51 (27.4)	26 (23.4)	60 (40.3)	190 (33.3)	11.4 (3)		.01	
	Discord	21 (16.8)	29 (15.6)	29 (26.1)	55 (36.9)	134 (23.5)	28.7 (3)		<.001	
	Twitch	20 (16)	27 (14.5)	8 (7.2)	29 (19.5)	84 (14.7)	3.2 (3)		.36	
	Tumblr	19 (15.2)	17 (9.1)	8 (7.2)	43 (28.9)	87 (15.2)	32.3 (3)		<.001	

^a^n=100 cisgender bisexual girls and n=11 cisgender bisexual boys.

^b^n=42 for transgender boys, n=53 for transgender girls, and n=54 for gender nonbinary adolescents.

^c^N/A: not applicable.

### Time Spent on LGBT Social Media Sites

Table 1 shows the detailed social media platforms used by our respondents. The most popular social media platforms for LGBT adolescents in this study were Instagram (483/571, 84.6%) and TikTok (451/571, 79%), followed by Facebook (377/571, 66%), Snapchat (Snap, Inc; 370/571, 64.8%), and Twitter (326/571, 57.1%). Approximately one-third (161/571, 28.2%) of them have used a platform that is specifically for LGBT populations. Platforms such as Discord (Discord, Inc), Twitch (Twitch Interactive, a subsidiary of Amazon.com, Inc), and Tumblr (Tumblr, Inc) were used by only a minority of adolescents. Significant general social media platform differences were found across LGBT subgroups; for example, transgender and gender nonbinary adolescents were less likely to use TikTok and Facebook and more likely to use Tumbler compared with lesbian and gay teenagers (all *P*<.001). The respondents spent an average of 3.6 (SD 2.7) hours per day on the LGBT-specific social media sites. The results of the MANOVA (Table 2) indicated that cisgender lesbian girls spent the most time per day on LGBT social media sites than all other groups, followed by gay boys, bisexual adolescents, and transgender or nonbinary adolescents (all *P*<.001). Correlational analyses yielded no significant associations between time spent on LGBT social media sites and other demographics (Table 3).

**Table 2:** Means, SDs, univariate results for adolescents’ social media experiences and mental health by lesbian, gay, bisexual, and transgender or nonbinary (LGBT) identities.

	Cisgender lesbian (n=125), mean (SD)	Cisgender gay (n=186), mean (SD)	Cisgender bisexual (n=111), mean (SD)	Transgender or nonbinary (n=149), mean (SD)	Total (N=571), mean (SD)	Univariate test results	
						*F test* (*df*)	*P* value
Social media LGBT discrimination	1.9 (1)	1.8 (1)	1.4 (0.9)	2.0 (1.2)	1.8 (1)	6.24 (*3*, *550*)	<.001
Time spent on LGBT social media sites	26.5 (22.6)	18.3 (14.7)	14.0 (19.9)	11.6 (10.3)	17.5 (17.8)	15.51 (*3*, *550*)	<.001
LGBT social justice civic engagement	2.2 (0.9)	2.1 (1)	1.6 (1.2)	2.0 (1.2)	2.0 (1.1)	6.08 (*3*, *550*)	<.001
Web-based social support	3.7 (0.8)	3.6 (0.8)	3.4 (1.0)	3.7 (1)	3.6 (0.9)	2.26 (*3*, *550*)	.08
Depressive symptoms	9.4 (7.0)	8.7 (7.1)	13.8 (6.7)	16.4 (6)	11.8 (7.5)	30.3 (*3*, *550*)	<.001
Anxiety symptoms	7.6 (5.8)	7.1 (5.9)	11.0 (5.6)	13.2 (5.6)	9.5 (6.3)	26.8 (*3*, *550*)	<.001
Substance use risk	2.3 (2.3)	1.9 (2.2)	2.1 (2.2)	1.4 (2)	1.9 (2.2)	4.7 (*3*, *550*)	.003

**Table 3:** Pearson product moment correlations among online variables, mental health, substance use, and selected demographics.

			Time spent on LGBT^a^ social media sites		LGBT social justice civic engagement		Social media LGBT discrimination		Web-based social support		Depressive symptoms	Anxiety symptoms		Substance use risk		Parent education		Financial insecurity		Disclosure on LGBT social media sites
**Time spent on LGBT social media sites**																				
	*r*	1		—^b^		—		—		—		—	—		—		—		—	
	*P* value	—		—		—		—		—		—	—		—		—		—	
**LGBT social justice civic engagement**																				
	*r*	0.2		1		—		—		—		—	—		—		—		—	
	*P* value	<.001		—		—		—		—		—	—		—		—		—	
**Social media LGBT discrimination**																				
	*r*	0.2		0.6		1		—		—		—	—		—		—		—	
	*P* value	.002		<.001		—		—		—		—	—		—		—		—	
**Web-based social support**																				
	*r*	0.09		0.3		0.2		1		—		—	—		—		—		—	
	*P* value	<.001		<.001		<.001		—		—		—	—		—		—		—	
**Depressive symptoms**																				
	*r*	0.2		0.2		0.4		−0.01		1		—	—		—		—		—	
	*P* value	<.001		<.001		<.001		0.81		—		—	—		—		—		—	
**Anxiety symptoms**																				
	*r*	0.2		0.2		0.4		−0.05		0.8		1	—		—		—		—	
	*P* value	<.001		<.001		<.001		.19		<.001		—	—		—		—		—	
**Substance use risk**																				
	*r*	0.08		0.3		0.2		0.07		0.1		0.1	1		—		—		—	
	*P* value	.05		<.001		<.001		.08		.003		.002	—		—		—		—	
**Parent education**																				
	*r*	−0.02		0.08		−0.02		0.2		−0.2		−0.2	0.05		1		—		—	
	*P* value	.63		.04		.63		<.001		<.001		<.001	0.23		—		—		—	
**Financial insecurity**																				
	*r*	−0.03		0.2		0.1		0.07		0.07		0.07	0.3		−0.1		1		—	
	*P* value	.36		<.001		.007		.06		.06		.06	<.001		.004		—		—	
**Disclosure on LGBT social media sites**																				
	*r*	0.1		0.3		0.2		0.2		0.1		0.1	0.1		0.001		0.02		1	
	*P* value	.02		<.001		<.001		<.001		.006		.006	.001		.99		.63		—	

^a^LGBT: lesbian, gay, bisexual, and transgender or nonbinary.

^b^Not applicable.

### Social Media LGBT Social Justice Civic Engagement

Only 6.3% (36/571) of youths did not engage in any web-based LGBT civic engagement; on average, they reported that they spent half their time on social media engaged in social justice activities (Table 2). The results of the MANOVA indicated that bisexual adolescents reported significantly lower frequencies of civic engagement than the other groups (all *P*<.001). Adolescents who had disclosed their identities to all or most people on LGBT-specific social media sites (mean 2.3, SD 1.0) reported more civic engagement than those who were out to a few people (mean 1.7, SD 1.0) or not out at all (mean 1.2, SD 1.2; *P* values <.001). Correlational analyses yielded a positive association between social media LGBT social justice civic engagement and financial insecurity, suggesting that individuals who experience financial insecurity may be less likely to engage in LGBT social justice activities on social media (Table 3).

### Exposure to Social Media LGBT Discrimination

Most youths (556/571, 97.4%) reported at least 1 instance of exposure to anti-LGBT web-based discrimination during the past month, and on average, they reported experiencing web-based discrimination about half the time they were on social media. The results of the MANOVA (Table 2) with Tukey post hoc comparison indicated that bisexual adolescents had significantly less exposure to web-based discrimination than the other groups (all *P*<.001). In contrast, transgender or nonbinary youth had significantly higher levels of exposure than others (all *P*<.001). Non-Hispanic White adolescents reported significantly higher levels of exposure to web-based discrimination (mean 1.9, SD 1.1) than Hispanic or Latinx adolescents (mean 1.5, SD 1.0; *P*=.01). Adolescents who had disclosed their identities to most or all people (mean 2.0, SD 1.0) on LGBT social media sites reported significantly higher levels of exposure to web-based discrimination than those who had disclosed their identities to only a few people (mean 1.6, SD 1.0; *P*=.005) or nobody (mean 1.4, SD 1.1; *P*<.001). No age differences were observed between the groups (*P*=.69-.99). Consistent with hypothesis 1, the results of the correlation analysis (Table 3) found that time spent on LGBT social media sites and LGBT social justice civic engagement were positively associated with exposure to LGBT web-based discrimination, indicating that individuals who engage in LGBT social media sites and LGBT social justice activities on social media may be more likely to experience discrimination.

### Web-Based Social Support on Social Media

The results of the MANOVA (Table 2) with Tukey post hoc comparison indicated no differences in web-based social support based on LGBT identities. Those who did not disclose their identity on the web (mean 3.0, SD 1.1) reported significantly less support than those who did (mean 3.6, SD 0.8) or those who disclosed to most or everyone (mean 3.7, SD 0.9; all *P*<.001). No racial, ethnic, or age differences were observed (*P*=.18-.99). The correlation analysis (Table 3) indicated that higher parental education was associated with more web-based social support. Partially consistent with the first hypothesis, web-based social support was positively associated with LGBT justice civic engagement but unrelated to the time spent on LGBT social media sites.

### Mental Health and Substance Use Risk

The results of the MANOVA indicated that transgender or nonbinary adolescents reported the highest risk of depressive and anxiety symptoms than all groups, followed by bisexual adolescents who had significantly higher risk than lesbian girls and gay boys (Table 2). However, lesbian girls and bisexual adolescents had a significantly higher substance use risk than transgender or nonbinary youth. Black adolescents reported significantly less depressive (mean 9.1, SD 7.3) and anxiety (mean 7.4, SD 6.0) symptoms than non-Hispanic White youth (mean 14.1, SD 7.6 for depressive symptoms; mean 10.4, SD 6.1 for anxiety symptoms; *P* values <.001). As shown in Table 3, higher parental education levels were associated with fewer depressive and anxiety symptoms. Financial insecurity was associated with a higher risk of substance use. Adolescents’ disclosure status on social media was unrelated to their mental health and substance use risk. Partially consistent with hypothesis 2, depressive and anxiety symptoms and substance use risk were positively associated with exposure to discrimination but were unrelated to social support on social media (Table 3).

**Figure 2:**
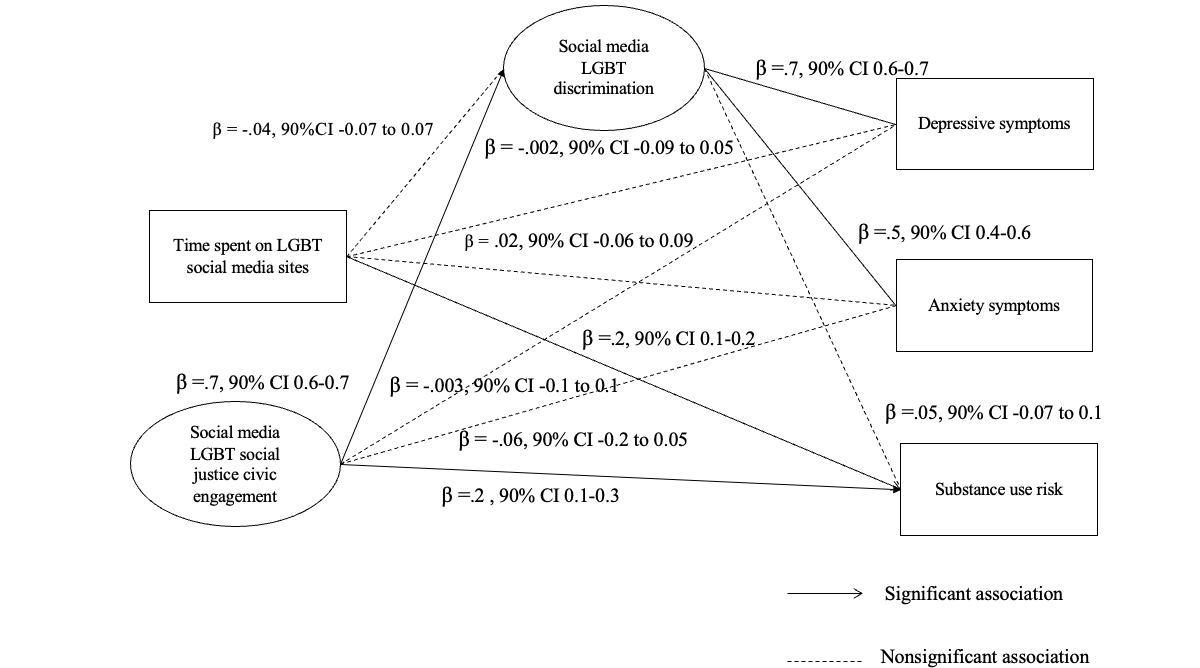
Results of the structural equation modeling analysis testing the mediating role of exposure to web-based discrimination in the positive association between time spent on lesbian, gay, bisexual, and transgender or nonbinary (LGBT) social media sites and social media social justice civic engagement with mental health and substance use.

To test hypothesis 3 with SEM analysis (refer to Figure 2 for results), latent variables included social media LGBT social justice civic engagement and exposure to web-based discrimination. Covariates included demographic factors associated with the studied variables, including age, gender and sexual identity, race or ethnicity, parent education level, financial insecurity, and whether they were out on LGBT social media sites. The model had an adequate fit (CFI=0.92; TLC=0.90; RMSEA=0.06; 90% CI 0.06-0.07). Partially consistent with the hypothesis, the model found that web-based discrimination fully explained the association between social media LGBT justice civic engagement with both depressive (β=.3, 90% CI 0.2-0.4) and anxiety symptoms (β=.3, 90% CI 0.2-0.4). However, it did not explain the positive direct association between social media LGBT justice civic engagement and substance use risk (β=.2, 90% CI 0.1-0.3), suggesting that individuals who engage in social justice activities on social media may be more likely to engage in substance use. Moreover, although bivariate correlational analysis yielded significant negative association between time spent on LGBT social media and depressive symptoms, there were no significant direct nor indirect associations between time spent on LGBT social media sites and mental health after accounting for LGBT justice civic engagement on social media in the SEM model. There was a significant direct association between time spent on LGBT social media sites and substance use risk (β=.2, 90% CI 0.1-0.2). An alternative SEM model tested whether higher levels of exposure to web-based discrimination were associated with more depressive symptoms, which in turn was associated with more LGBT social justice civic engagement on social media and more time spent on LGBT social media sites. Results yielded an inadequate fit (CFI=0.87; TLI=0.85; RMSEA=0.09; 90% CI 0.08-0.09), with a nonsignificant indirect association between exposure to discrimination and civic engagement on social media through depressive symptoms (β=.01, 90% CI −0.001 to 0.02).

**Figure 3:**
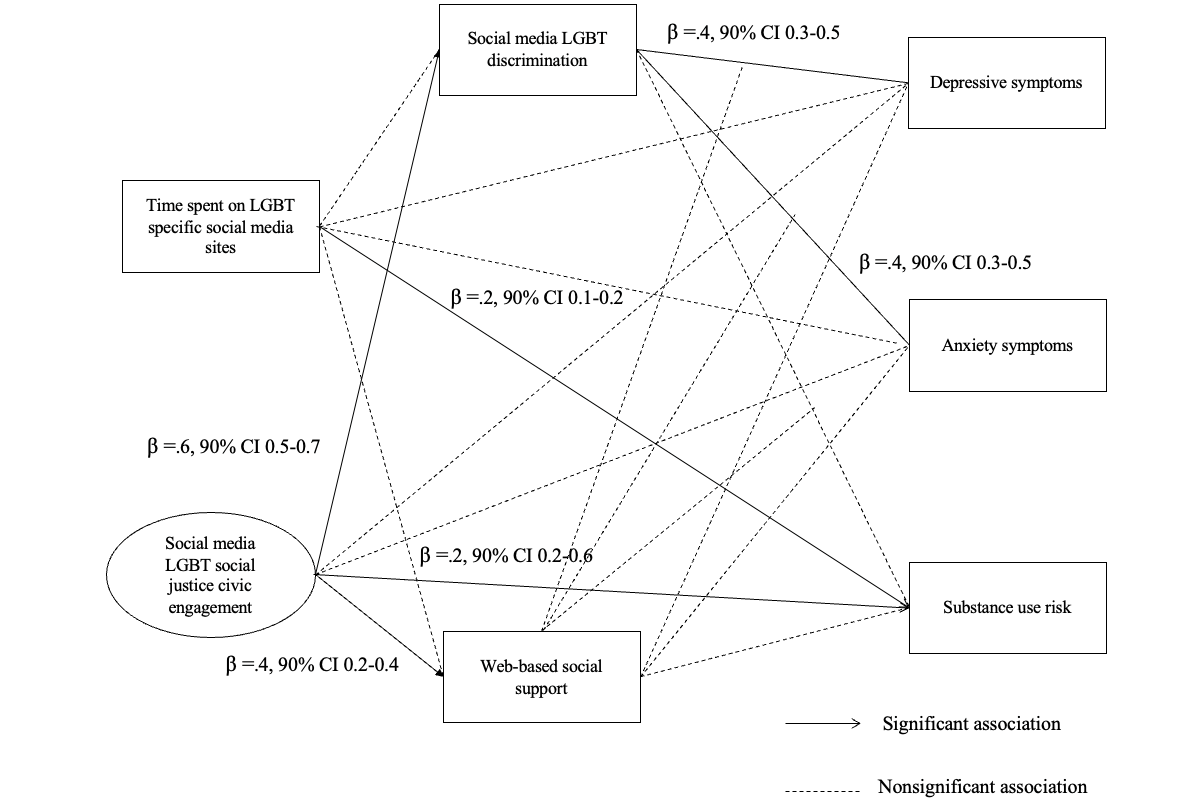
Results of the structural equation modeling analysis testing the moderating role of web-based social support in the positive association between exposure to social media lesbian, gay, bisexual and transgender or nonbinary (LGBT) discrimination and mental health and substance use. Nonsignificant results were omitted for presentation.

To test hypothesis 4 (Figure 3), we included standardized *z* scores of exposure to web-based discrimination and social support and an interaction term between the 2 variables. The only latent variable in this model was social media LGBT social justice civic engagement, and the same covariates were included. The results yielded an adequate fit (CFI=0.95; TLI=0.90; RMSEA=0.06; 90% CI 0.06-0.07). Social media civic engagement was associated with greater social support from LGBT friends on social media (β=.4, 90% CI 0.2-0.4). However, web-based social support was unrelated to depressive symptoms (90% CI −0.7 to 0.6), anxiety (90% CI −1.0 to 0.1), and substance use risk (90% CI −0.2 to 0.2). Contrary to our hypothesis, it did not moderate the association between social media civic engagement and associated exposure to web-based discrimination with depressive (90% CI −0.07 to 0.1) and anxiety symptoms (90% CI −0.06 to 0.1) and substance use (90% CI −0.04 to 0.01).

## Discussion

### Principal Findings

Our study adds to the small but growing literature on the relationship between social media engagement and mental health among LGBT adolescents [2,3,7,9] by applying the minority stress and stress-buffering hypotheses [19,21]. Specifically, we examined the relationships among participation on LGBT-specific media sites, web-based social support, exposure to heterosexist and transphobic discriminatory posts, and mental health and substance use. We observed that the time spent on LGBT sites was associated with both increased exposure to discrimination and social support. Exposure to anti-LGBTQ posts may be a function of social media algorithms sending both LGBT positive and negative posts to individuals who have shown “interest” in LGBT topics [41]. Compared with previous research on experiences of cyberbullying rates among LGBT adolescents [42], in this study, 97.4% (556/571) of the youths reported at least 1 incident of web-based discrimination. One possible explanation for this is that most respondents (519/571, 90.9%) had made their sexual and gender identity visible on the web to at least a select few persons, and the more people they were out to on the web, the more they were associated with increased exposure to discrimination. Transgender and gender nonbinary youth and non-Hispanic White youth reported the highest level of web-based discrimination; however, transgender or nonbinary youth were disproportionately represented among White respondents, which may account for the effect of ethnicity. Increased exposure of transgender or nonbinary youth to transphobic web-based content may be a function of recent bans of transgender youth from certain schools and competitive sports and state legislatures banning puberty blockers and other gender-affirming care [43,44].

Prior research on social media community engagement among LGBT youth has only used time of use as the unit of analysis and has highlighted the positive impact of LGBT community engagement [2,3,7]. However, in this study, although the time spent on LGBT social media sites was related to fewer depressive symptoms in the correlation analysis, the potential benefits became statistically insignificant after social media civic engagement was accounted for. These findings highlight the importance of examining the nature of the activities that LGBT youth engage in web-based communities. The finding that LGBT civic engagement increases exposure to discrimination is consistent with past research on social media racial justice civic engagement among adolescents from racial and ethnic minoritized groups [12]. The nature of social media civic engagement may explain the increased exposure to discriminatory posts because the goal of these activities is to combat heterosexist and transphobic incidents or policies by sharing posts related to addressing LGBT injustices [18] and coordinating activities to promote ways to combat these injustices [17]. In addition, the potential impact of state laws preventing the discussion of any material related to the lives and rights of LGBT youth s in schools during the winter of 2022 may also contribute to the increased likelihood of youth being exposed to discrimination on social media [43,44]. This may also explain why social support is higher for those involved in civic engagement, because many items on the Social Support Scale assessed youth’s desire to offer and receive support within the context of hurtful messaging. Moreover, youth who never disclosed their identity on the web reported significantly less support than those who did.

Not only did web-based civic engagement increase exposure to heterosexist and transphobic content, but in line with minority stress theory [19], SEM analyses indicated that such exposure completely accounted for the association between web-based social justice civic involvement and symptoms of depression and anxiety. Strengthening the conclusions regarding the hypothesized relationship between civic engagement depression and mental health, we found an inadequate fit when testing an alternative model testing the hypothesis that higher levels of exposure to web-based discrimination were associated with more depressive symptoms, which in turn led to greater LGBT social justice civic engagement. Transgender or nonbinary adolescents reported the highest risk of depressive and anxiety symptoms among all groups, which is consistent with the higher levels of web-based discrimination they reported. Black adolescents reported significantly fewer depression and anxiety symptoms than other youth. This is contrary to recent reports placing adolescents from racial and ethnic minoritized groups at higher levels of mental health risk [45]. Previous literature suggests that this may be related to the Black-White depression paradox, which is the lower prevalence of major depression among non-Hispanic Black individuals despite their greater exposure to major life stressors [46]. Possible unique protective factors among Black youth have been proposed, such as family and community support and cultural resilience, but a recent review found limited evidence in support of any of the proposed mechanisms [47]. Another possible explanation is that the measures did not fully capture the unique experiences and expressions of depression and anxiety among Black adolescents. Future research should explore the underlying mechanisms that may account for this paradox and investigate how intersecting identities, such as race or ethnicity and LGBT identities, may affect the experience of mental health issues among youth.

Our finding that social media civic engagement was directly associated with substance use is consistent with research with adolescents in general, which has shown that more time spent on social media is associated with higher substance use risk and positive associations between offline activism and substance use [17,48-50]. Contrary to our hypotheses, exposure to web-based discrimination did not explain the significant association between time spent on LGBT social media sites generally and LGBT social justice civic activities, specifically with increased substance use risk. However, cisgender lesbian girls and bisexual adolescents (mostly girls) were at a significantly higher substance use risk than other youth in our study. The results are in accordance with research indicating that LGBT cisgender girls report significantly more substance use risk than boys [51-53] but are inconsistent with prior research indicating higher substance use risk among transgender or nonbinary youth.

Contrary to our hypothesis, our data did not support the stress-buffering model [21-23,25]. Web-based social support among LGBT friends was unrelated to mental health and did not mitigate the negative impact of web-based discrimination on mental health. Prior research on the mitigating effect of social support on exposure to discrimination has produced mixed findings. Some studies involving LGBT adolescents support the stress-buffering hypothesis [17,20], whereas a recent meta-analysis indicates that web-based social support from acquaintances on the internet was only related to self-esteem but not depression [18], and research comparing the impact of web-based and offline social support indicates that only offline social support was associated with better mental health [19]. The positive association between web-based social support and exposure to web-based discrimination in this study may explain such differences. These findings indicate that the advantages of receiving support through social media could be short-term and passing, whereas the adverse effects stemming from encountering discriminatory content on social media may have a more enduring impact.

### Limitations and Future Directions

Although our study included an ethnically diverse sample of LGBT youth, our recruitment procedures primarily relied on Instagram posts in LGBT communities, and inclusion criteria identified frequent social media users (youth who spent more than 5 days per week on social media). Thus, our sample was limited to those who frequently engage in those web-based communities and who are more likely to reveal their LGBTQ identity on the web and be more susceptible to web-based discrimination. Similarly, although our sample recruited youth from 48 of the 50 US states, our results reflect LGBT adolescents’ experiences in the United States and cannot be generalized to other national contexts. Relatedly, the small sample size of respondents who self-identified as questioning or unsure and cisgender gay may limit the generalizability of our findings to these subgroups, and the unbalanced sample sizes may have limited our ability to explore potential racial and ethnic differences in exposure to LGBT web-based discrimination. Our data collection strategies may also have some limitations. For example, most survey items required a forced response to prevent missing data, which may have influenced participants who chose to drop out of the survey, although this was only a small percentage of respondents (7.7%). An additional limitation is the self-reported nature of the data, which may be subject to social desirability, memory bias, and inaccuracies. Furthermore, the cross-sectional nature of our data precludes us from making causal inferences, and future research should consider longitudinal designs to test the temporal associations between the variables examined. There are other important factors that influence mental health and substance use risks that were not examined in this study. For instance, future studies should examine LGBT youth’s in-person experiences with heterosexism and transphobia across school, family, peer, and other contexts and examine how in-person and web-based discrimination independently and conjointly influence mental health. In addition, our measures may not fully capture the unique experiences and expressions of depression and anxiety among Black adolescents. To examine the intersectional experiences of LGBT adolescents from racial and ethnic minoritized groups, future studies should include enough participants from both racial and ethnic and LGBT subgroups as well as ask culturally sensitive questions particularly targeted to one’s experience as a LGBT youth with specific racial and ethnic identities.

### Conclusions

Engaging in LGBT web-based communities is prevalent among LGBT adolescents. This study highlights that the nature of web-based activities can increase both social support and exposure to heterosexist and transphobic content. Engaging in LGBT social justice web-based activities can increase discrimination and mental health risks unmitigated by social support. Offline civic engagement has been associated with positive mental health among LGBT youth [17]. However, these offline activities can be more insular, allowing youth to engage with other LGBT members without unrestricted exposure to anti-LGBT anonymous others experienced on social media, thereby increasing the moderating effect of social support on offline discriminatory experiences. However, nationally, LGBT youth have greater access to civic engagement activities on social media than may be available to them offline. The results of this study thus call for social media policy changes to mitigate the effects of algorithms that expose youth to heterosexist and transphobic messaging. These include strategies such as adopting machine learning algorithms that can recognize and remove harmful content more efficiently and providing clear and accessible reporting mechanisms for users to report discriminatory or harmful content. Youth can also benefit from research applying machine learning tools to identify mental health or substance use concerns [54] and to increase the effectiveness of web-based social support to mitigate the effect of discrimination on the mental health of LGBT youth. Such tools can be extended to the development of web-based support groups or interventions that incorporate peer support into mental health treatment for LGBT youth. Finally, training programs for mental health professionals should include methods for helping LGBT youth identify and address the impact of web-based discrimination and stigma and draw on social support to enhance the positive experience of social justice civic engagement.

## Appendix

Multimedia Appendix 1 Confirmatory factor analysis results.

## Abbreviations

CFI: comparative fit index
GAD-7: 7-item Generalized Anxiety Disorder Screener
LGBT: lesbian, gay, bisexual, and transgender or nonbinary
MANOVA: multivariate analysis of variance
PHQ-A: Patient Health Questionnaire modified for Adolescents
RMSEA: root mean square error of approximation
SEM: structural equation modeling
TLI: Tucker-Lewis Index
