# Supplementary material for: Associations Among Web-Based Civic Engagement and Discrimination, Web-Based Social Support, and Mental Health and Substance Use Risk Among LGBT Youth: Cross-Sectional Survey Study
Source: J Med Internet Res. 2023 Jun 26;25:e46604. doi: 10.2196/46604 (PMC10337473; doi:10.2196/46604)
Supplement: Multimedia Appendix 1 [file jmir_v25i1e46604_app1.doc]

Appendix 1 – Confirmatory Factor Analysis Results.

*LGBTQ+ Social Justice Civic Engagement*

Two confirmatory factor analyses on the revised scale were conducted to compare the two-factor model (CFI = .98, TLC = .97, RMSEA = .08, 90%CI [.06, .10]) with the one-factor model (CFI = .95, TLC = .92, RMSEA = .14, 90%CI [.12, .16]). Both models had adequate fit, yet the civic publication and activity coordination subscales were highly correlated with each other (r = 0.9, p < .001), thus we adopted the one-factor model and allowed covariates between items 1 (“Post/share links…”) and 2 (“Post/share photos…”) given their high correlated errors (MI = 40.1). The final one-factor model had good fit (CFI = .96, TLC = .94, RMSEA = .12, 90%CI [.10, .14]), with factor loadings ranged from .79 to .86.

*Social Media LGBTQ+ Discrimination*

Two confirmatory factor analyses on the revised scale were conducted to compare the two-factor model (CFI = .99, TLC = .98, RMSEA = .07, 90%CI [.05, .09]) with the one-factor model (CFI = .91, TLC = .87, RMSEA = .16, 90%CI [.14, .18]). Although the one-factor model did not have adequate fit, the personal and vicarious discrimination subscales were highly correlated with each other (r = 0.7, p < .001). Thus, we adopted the one-factor model and allowed covariates between items 4 (“People have said things that were untrue on social media about people of my LGBTQ+ identity”) and 5 (“I have witnessed people saying mean or rude things on social media about another person’s LGBTQ+ identity”) given their high correlated errors (MI = 73.6). The final one-factor model had adequate fit (CFI = .94, TLC = .91, RMSEA = .12, 90%CI [.10, .14]), with factor loadings ranged from .55-.83.

*Social Support on LGBTQ+ Social Media*

Two confirmatory factor analyses were conducted for the revised scale to compare the two-factor model (CFI = .98, TLC = .97, RMSEA = .07, 90%CI [.05, .09]) with the one-factor model (CFI = .91, TLC = .87, RMSEA = .14, 90%CI [.12, .16]). Although the initial analysis of the one-factor model did not yield an adequate fit, offering and receiving support subscales were highly correlated with each other (r = 0.8, p < .001). For the one-factor model, significant correlated errors (MI = 61.3) emerged between item 1 (i.e., “If I see some LGBTQ+ friends post a social media status update that indicates they are upset, I try to post a comforting comment on their status”) and 2 (i.e., “It is important to me to try to cheer up my LGBTQ+ friends by commenting on their social media status updates when it appears that they feel distressed”), thus we allowed such covariates in the next CFA model, which yielded an adequate fit (CFI = .95, TLC = .91, RMSEA = .11, 90%CI [.10, .13]).
